# Effects of the sigma-1 receptor agonist blarcamesine in a murine model of fragile X syndrome: neurobehavioral phenotypes and receptor occupancy

**DOI:** 10.1038/s41598-021-94079-7

**Published:** 2021-08-25

**Authors:** Samantha T. Reyes, Robert M. J. Deacon, Scarlett G. Guo, Francisco J. Altimiras, Jessa B. Castillo, Berend van der Wildt, Aimara P. Morales, Jun Hyung Park, Daniel Klamer, Jarrett Rosenberg, Lindsay M. Oberman, Nell Rebowe, Jeffrey Sprouse, Christopher U. Missling, Christopher R. McCurdy, Patricia Cogram, Walter E. Kaufmann, Frederick T. Chin

**Affiliations:** 1 Department of Radiology, Stanford University, Stanford, CA 94305 USA; 2 FRAXA-DVI, FRAXA, Santiago, Chile; 3 IEB, Faculty of Science, University of Chile, Santiago, Chile; 4 Fraunhofer Chile Research, Center for Systems Biotechnology, Santiago, Chile; 5 Faculty of Engineering and Business, Universidad de las Américas, Santiago, Chile; 6 Anavex Life Sciences Corp., New York, NY 10019 USA; 7 Center for Neuroscience & Regenerative Medicine, Uniformed Services University of the Health Sciences, Bethesda, MD USA; 8 Department of Medicinal Chemistry, College of Pharmacy, University of Florida, Gainesville, FL 32610 USA; 9 Department of Human Genetics, Emory University School of Medicine, Atlanta, GA 30322 USA

**Keywords:** Pharmacology, Target validation, Positron-emission tomography, Small molecules, Target validation, Cognitive neuroscience, Molecular neuroscience, Preclinical research, Translational research, Molecular medicine

## Abstract

Fragile X syndrome (FXS), a disorder of synaptic development and function, is the most prevalent genetic form of intellectual disability and autism spectrum disorder. FXS mouse models display clinically-relevant phenotypes, such as increased anxiety and hyperactivity. Despite their availability, so far advances in drug development have not yielded new treatments. Therefore, testing novel drugs that can ameliorate FXS’ cognitive and behavioral impairments is imperative. ANAVEX2-73 (blarcamesine) is a sigma-1 receptor (S1R) agonist with a strong safety record and preliminary efficacy evidence in patients with Alzheimer’s disease and Rett syndrome, other synaptic neurodegenerative and neurodevelopmental disorders. S1R’s role in calcium homeostasis and mitochondrial function, cellular functions related to synaptic function, makes blarcamesine a potential drug candidate for FXS. Administration of blarcamesine in 2-month-old FXS and wild type mice for 2 weeks led to normalization in two key neurobehavioral phenotypes: open field test (hyperactivity) and contextual fear conditioning (associative learning). Furthermore, there was improvement in marble-burying (anxiety, perseverative behavior). It also restored levels of BDNF, a converging point of many synaptic regulators, in the hippocampus. Positron emission tomography (PET) and ex vivo autoradiographic studies, using the highly selective S1R PET ligand [^18^F]FTC-146, demonstrated the drug’s dose-dependent receptor occupancy. Subsequent analyses also showed a wide but variable brain regional distribution of S1Rs, which was preserved in FXS mice. Altogether, these neurobehavioral, biochemical, and imaging data demonstrates doses that yield measurable receptor occupancy are effective for improving the synaptic and behavioral phenotype in FXS mice. The present findings support the viability of S1R as a therapeutic target in FXS, and the clinical potential of blarcamesine in FXS and other neurodevelopmental disorders.

## Introduction

Fragile X Syndrome (FXS) is the most common inherited neurodevelopmental disorder, affecting approximately 1/4000 males and 1/6000–1/8000 females in the United States^1^. FXS results from a trinucleotide expansion of a CGG repeat in the 5′ untranslated region of the fragile X mental retardation 1 (*FMR1*) gene on the X chromosome, that leads to atypical gene methylation and transcriptional silencing with the consequent reduction in the synthesis of the gene product (i.e., fragile X mental retardation protein or FMRP). Larger expansions (> 200 repeats) result in gene silencing and are termed full mutation or simply FXS^1^, while smaller expansions (55–200 repeats) are termed premutation. The latter are not associated with gene silencing but with messenger ribonucleic acid (mRNA) accumulation and other neurologic and endocrine phenotypes^1^.

Individuals with FXS are at increased risk of cognitive impairment, characteristic physical features, and behavioral abnormalities such as anxiety, hyperarousal, attention-deficit/hyperactivity disorder (ADHD) features, self-injurious behavior, aggression, irritability, and stereotypic and perseverative behavior^2^. Additionally, a large proportion of individuals with FXS display autistic features, with 20–50% meeting diagnostic criteria for autism spectrum disorder (ASD)^2–4^. Levels of FMRP correlate with the overall severity of the FXS phenotype, with males being more severely affected due to the X-linked pattern of the disorder^5–7^. As other neurodevelopmental disorders, FXS is considered a synaptic disorder with characteristic deficits in long-term potentiation and in homeostatic plasticity as well as increases in long-term depression due to excessive group I metabotropic glutamate receptor activity^1,8,9^. Multiple drug trials targeting the excitatory-inhibitory imbalances linked to these synaptic abnormalities have been largely unsuccessful in children and adults with FXS^10,11^. Other targets investigated in FXS include serotonin, metabotropic glutamate receptor (mGluR) and the gamma-aminobutyric acid (GABA) system^1,12^. New therapeutic strategies are needed, which may come from targeting cellular and synaptic homeostasis and multiple signaling pathways.

The sigma-1 receptor (S1R) is an intracellular chaperone protein located at the endoplasmic reticulum-mitochondria interface, which plays important roles in inter-organelle communication and cellular response to stress. S1R activation regulates calcium signaling from the endoplasmic reticulum to the mitochondria, improving mitochondrial function and decreasing levels of reactive oxygen species (ROS). Other cellular processes implicated in many neurodevelopmental and neurodegenerative disorders and modulated by S1Rs include proteostasis/autophagy and neuroinflammation^13–16^. Of relevance to synaptic disorders like FXS, there is increasing evidence that calcium influx into mitochondria regulates key synaptic processes such as firing rate set point (e.g., hippocampus) and homeostatic synaptic plasticity^17^. Indeed, administration of a S1R agonist has corrected maladaptive homeostatic synaptic scaling in a mouse model of Huntington disease^18^. Further support for a S1R-based intervention in FXS comes from receptor distribution studies in brain^19^. Areas of relatively higher S1R density include the hippocampus (pyramidal, non-pyramidal layers, granular layer of the dentate gyrus), septum, paraventricular nucleus of the hypothalamus, anterodorsal thalamic nucleus, dorsal raphe, substantia nigra, locus coeruleus, and cerebellum. Considering this widespread pattern of distribution and previous experience in relevant mouse models^20,21^, S1R agonists have the potential to correct defective synaptic mechanisms and enhance compensatory processes in multiple brain regions.

FMRP deficiency is linked to multiple cell signaling abnormalities, including the adenylate cyclase/ protein kinase A (PKA)^22,23^, phosphatidylinositol 3-kinase/protein kinase B/mechanistic target of rapamycin (PI3K/Akt/mTOR)^24–26^, mitogen-activated protein kinase/extracellular signal-regulated kinase (MAPK/ERK)^25,26^, and glycogen synthase kinase 3 beta (GSK-3β)^27^ pathways, and to FMRP interacting regulatory complexes such as Ras-related C3 botulinum toxin substrate 1 (Rac1)-Wave^28,29^, which ultimately result in gene dysregulation and synaptic abnormalities^9^. A key pathway to which the aforementioned signaling and synaptic processes, known to be affected in FXS and other neurodevelopmental disorders, converge bidirectionally is brain-derived neurotropic factor (BDNF) signaling^30,31^. It is reported that changes in BDNF levels in rodent FXS models effect cognitive and sensorimotor deficits^32^. Modulation of BDNF levels by S1R activation, which has been observed in different neural systems and animal models of neurologic disorders^18,20,33^, may be of mechanistic importance for compensating multiple signaling and synaptic pathways.

Taking advantage of mouse models of FMRP deficiency, employed in mechanistic and translational studies, we report here on an initial preclinical assessment of tetrahydro-*N,N*-dimethyl-2,2-diphenyl-3-furanmethanamine hydrochloride (ANAVEX2-73, blarcamesine, half maximal inhibitory concentration, IC_50_ = 860 nM for the S1R^34^), a S1R agonist and muscarinic receptor modulator^34^ in FXS. Blarcamesine is currently tested in several clinical efficacy studies in Rett syndrome, Parkinson’s disease dementia and Alzheimer’s disease. Results to date demonstrate a highly favorable safety profile and dose-dependent cognitive and functional improvements, as assessed by the MMSE (Mini Mental State Examination) and the ADCS-ADL (Alzheimer’s Disease Cooperative Study-Activities of Daily Living), respectively^35^. Blarcamesine has also exhibited anticonvulsant, anti-amnesic, neuroprotective, and other compensatory effects in various animal models of neurologic disorders^34,36–38^. Of particular relevance is recent work in one of the most clinically relevant mouse models of Rett syndrome, a neurodevelopmental disorder that shares many neurobiological features with FXS, including abnormal BDNF signaling^30^. Blarcamesine yielded beneficial effects in multiple neurologic phenotypes covering sensory, motor, and autonomic features^21^, which included the distinctive hindlimb clasping that resembles the disorder’s hallmark hand stereotypies^39^.

The present study employs both *Fmr1* knockout (KO) 1 and KO2 mouse models of FXS^40–42^ and covers multiple aspects of blarcamesine’s action: an evaluation of three neurobehavioral paradigms representing key cognitive and behavioral aspects of the FXS phenotype, and parallel assessments of several key signaling pathways in a S1R-enriched region. An important element to this study is the inclusion of a 6-(3-[^18^F]fluoropropyl)-3-(2-(azepan-1-yl)ethyl)benzo[*d*]thiazol-2(3*H*)-one ([^18^F]FTC-146) positron emission tomography (PET) imaging component, in order to establish the S1R receptor occupancy of blarcamesine within the range of efficacious dosing and to expand the foundations of the drug’s effects on behavioral phenotypes. [^18^F]FTC-146 has been previously characterized^43^ as a highly selective S1R PET probe (sigma-1 receptor inhibitor constant, K_i_ = 2.5 × 10^–3^ nM; sigma-2 receptor K_i_ = 3.6 × 10^2^ nM) with demonstrated utility in mice, rats, monkeys and in the clinic^44–47^. In particular, the high specificity of [^18^F]FTC-146 has been validated using ex vivo autoradiography and immunostaining by establishing the relationship of radiotracer accumulation to S1R staining^46^. In turn, these findings in animal models have more recently been translated into first-in-human studies with clinical-grade [^18^F]FTC-146^47^. The incorporation of [^18^F]FTC-146 PET imaging into this study provides insight as to how blarcamesine and the S1R receptor interact in vivo. Taken together, the behavioral and biochemical results, supported by the S1R occupancy profile, demonstrate that blarcamesine is a potentially valuable therapeutic approach for patients with FXS.

## Results

### Blarcamesine substantially improved the behavioral phenotype of Fmr1 KO2 mice

**Figure 1:**
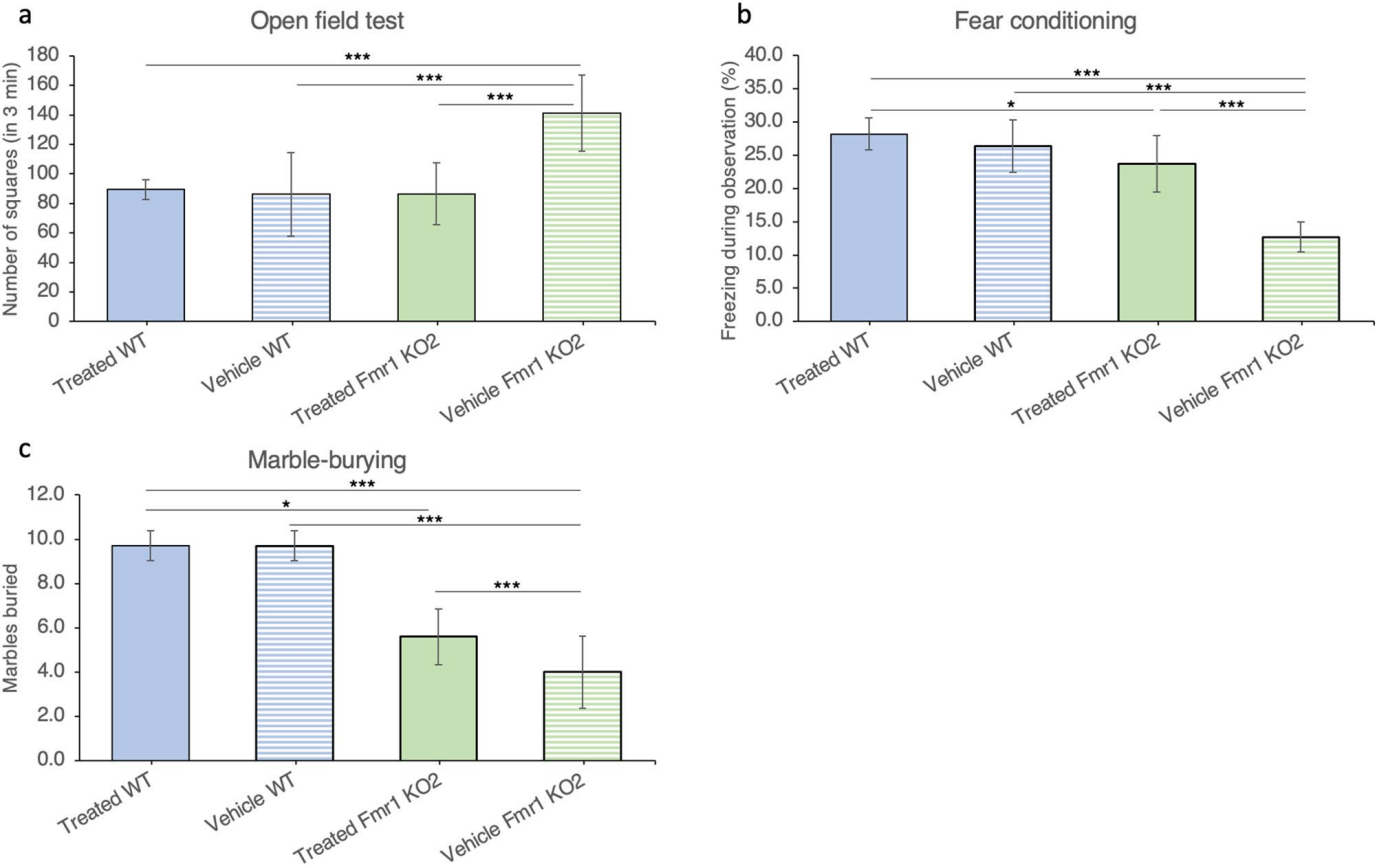
Behavior in *Fmr1* KO2 mice improves in three tested paradigms with chronic dosing of blarcamesine. (**a**) Open field paradigm. Twice-daily treatment with blarcamesine 1 mg/kg IP for 14 days normalized increased hyperactivity observed in vehicle-treated *Fmr1* KO2 mice (N = 10 per group; *Fmr1* KO2-Vehicle vs. WT-Vehicle, *Fmr1* KO2-Vehicle vs. WT-Treated, *Fmr1* KO2-Vehicle vs. *Fmr1* KO2-Treated: All ***p < 0.001; other comparisons not significant). (**b**) Fear conditioning paradigm. Twice-daily treatment with blarcamesine 1 mg/kg IP for 14 days normalized freezing behavior reduction observed in vehicle-treated *Fmr1* KO2 mice in the contextual fear paradigm (N = 10 per group; *Fmr1* KO2-Vehicle vs. WT-Vehicle, *Fmr1* KO2-Vehicle vs. WT-Treated, *Fmr1* KO2-Vehicle vs. *Fmr1* KO2-Treated: All ***p < 0.001; *Fmr1* KO2-Treated vs. WT-Treated: *p < 0.05; other comparisons not significant). (**c**) Marble burying paradigm. Twice-daily treatment with blarcamesine 1 mg/kg IP for 14 days significantly reduced the deficits in marble-burying behavior characteristic of vehicle-treated *Fmr1* KO2 mice (N = 10 per group; *Fmr1* KO2-Vehicle vs. WT-Vehicle, *Fmr1* KO2-Vehicle vs. WT-Treated: Both ***p < 0.001; *Fmr1* KO2- Vehicle vs. *Fmr1* KO2-Treated *p < 0.05; *Fmr1* KO2-Treated vs. WT-Vehicle ***p < 0.001; *Fmr1* KO2-Treated vs. WT-Treated ***p < 0.001; other comparisons not significant).

Comparisons between *Fmr1* KO2 groups demonstrated a significant reduction in total distance traveled (number of squares crossed in 3 min) by the blarcamesine-treated animals with respect to vehicle-treated mice (Student’s t-test p = 0.0006). The relevance of these changes was confirmed by comparing vehicle-treated *Fmr1* KO2 mice to their wild type (WT) counterparts, since the former displayed an increase in the abovementioned measure of hyperactivity (p < 0.001). When all 4 mouse groups were contrasted, chronic treatment with blarcamesine significantly reduced the behavior in *Fmr1* KO2 mice to levels indistinguishable from those observed in vehicle-treated WT mice (Fig. 1a). Since tests for equality of variances showed trend-level p-values (i.e., borderline equal variance among groups), nonparametric Kruskal–Wallis analysis of variance (ANOVA) and Wilcoxon rank sum posthoc tests were also performed. These confirmed the parametric ANOVA and posthoc test results.

In the contextual fear conditioning paradigm, similar differences between *Fmr1* KO2 groups were also found. There was a significant increase in percentage of freezing behavior in drug-treated *Fmr1* KO2 mice when compared with their vehicle-treated counterparts (Student’s t-test p < 0.0001). Again, the relevance of this effect was demonstrated by comparing vehicle-treated *Fmr1* KO2 mice to their WT counterparts, since the mutant mice displayed a significant decrease in freezing response (p < 0.001). The four-group analyses showed that the improvements in the blarcamesine-treated *Fmr1* KO2 mice were at the phenotype rescue level (i.e., no differences between drug-treated *Fmr1* KO2 mice and vehicle-treated WT animals) (Fig. 1b).

A species-specific behavior, which represents anxiety and perseverative behavior^48,49^, marble-burying activity was mildly increased (improved) by drug administration when vehicle- and blarcamesine-treated *Fmr1* KO2 groups were compared (Welch’s t-test p = 0.025). The 4-group comparison confirmed that vehicle-treated *Fmr1* KO2 mice buried significantly fewer marbles than similarly treated WT mice (p < 0.001). As in the paired t-test, the ANOVA’s posthoc test demonstrated that blarcamesine partially rescued this behavior in *Fmr1* KO2 animals (p ≤ 0.05) (Fig. 1c). Data for the WT were not normally distributed (leptokurtic), compatible with a ceiling effect. In line with this, tests for equality of variances showed borderline (trend level) comparable variances. Therefore, nonparametric tests were also performed. They confirmed the abovementioned parametric test results.

### Blarcamesine restores BDNF levels in the hippocampus of Fmr1 KO2 mice

In order to yield insight into the mechanisms underlying the behavioral improvements described above, we examined the levels of several key cell signaling markers in the hippocampus. As for the behavioral paradigms, we first analyzed the effects of blarcamesine by comparing vehicle- and drug-treated *Fmr1* KO2 mice.

**Figure 2:**
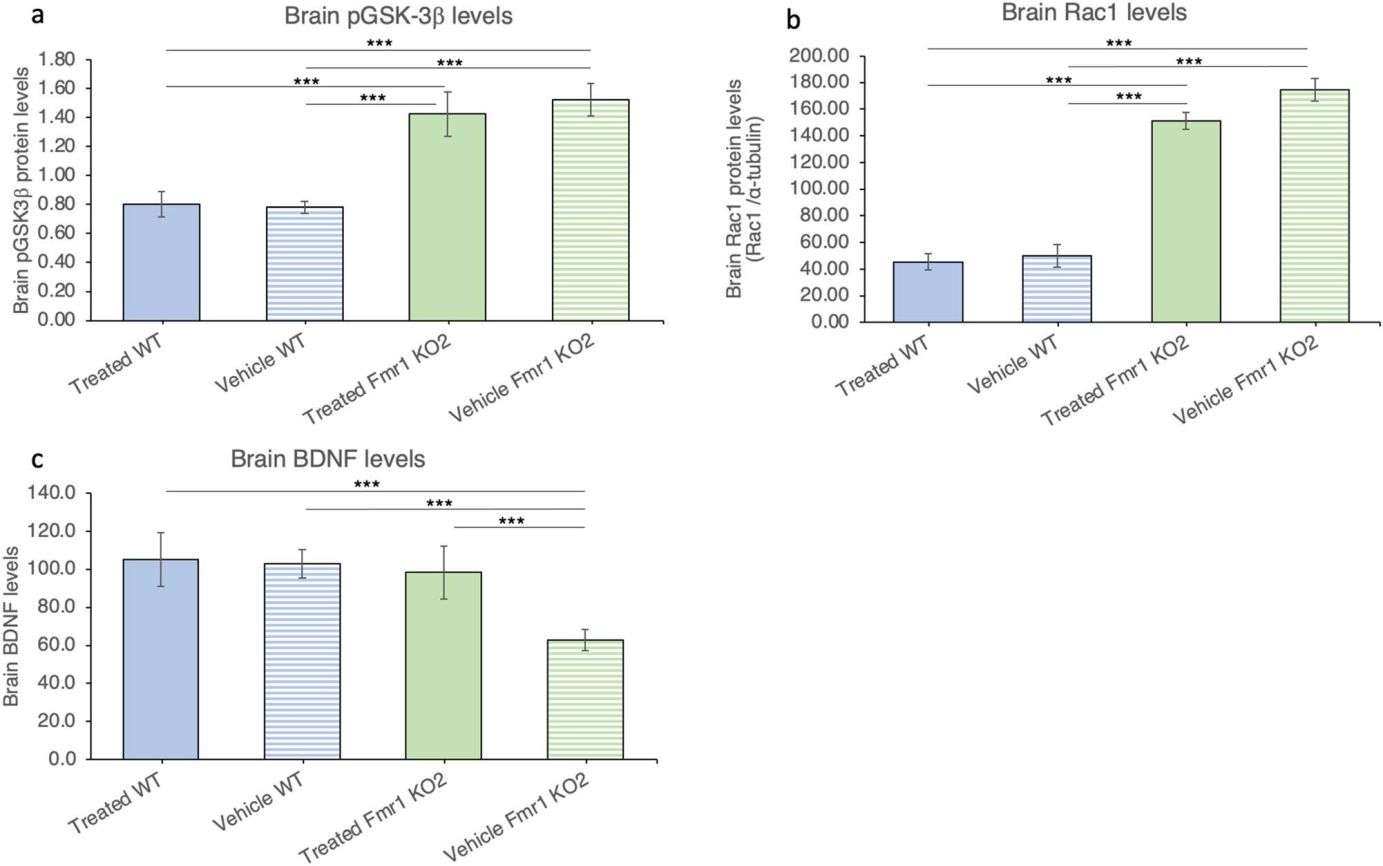
Fmr1 KO2 mouse brain BDNF levels recover in assays with use of blarcamesine chronic dosing (twice-daily treatment with blarcamesine 1 mg/kg IP for 14 days). (**a**) pGSK-3β levels in hippocampus. (N = 10 per group; *Fmr1* KO2-Vehicle vs. WT-Vehicle, *Fmr1* KO2-Vehicle vs. WT-Treated, *Fmr1* KO2-Treated vs. WT-Vehicle, *Fmr1* KO2-Treated vs. WT-Treated: All ***p < 0.001; *Fmr1* KO2-Vehicle vs. *Fmr1* KO2-Treated, WT-Vehicle vs. WT-Treated: Both not significant). (**b**) Rac1 levels in hippocampus. (N = 10 per group; *Fmr1* KO2-Vehicle vs. WT-Vehicle, *Fmr1* KO2-Vehicle vs. WT-Treated, *Fmr1* KO2-Treated vs. WT-Vehicle, *Fmr1* KO2-Treated vs. WT-Treated: All ***p < 0.001; *Fmr1* KO2-Vehicle vs. *Fmr1* KO2-Treated, WT-Vehicle vs. WT-Treated: Both not significant). (**c**) BDNF levels in hippocampus. (N = 6 per group; *Fmr1* KO2-Vehicle vs. WT-Vehicle, *Fmr1* KO2-Vehicle vs. WT-Treated, *Fmr1* KO2-Vehicle vs. *Fmr1* KO2-Treated: All ***p < 0.001; other comparisons not significant).

Phosphorylated GSK-3β (pGSK-3β) and Ras-related C3 botulinum toxin substrate 1 (Rac1) levels in vehicle-treated *Fmr1* KO2 mice were significantly higher than in vehicle-treated WT animals (both p < 0.0001). Administration of blarcamesine had no effect on the levels of these two signaling molecules in *Fmr1* KO2 mice (Fig. 2a,b).

As previously mentioned, BDNF constitutes a convergence point of PI3K/Akt/mTOR and MAPK/ERK pathways and other regulatory pathways^31,50,51^. BDNF levels in hippocampal homogenates in vehicle-treated *Fmr1* KO2 mice were significantly lower than in vehicle-treated WT animals (Welch’s t-test p < 0.05); comparisons between vehicle- and drug-treated *Fmr1* KO2 mice showed significantly higher BDNF levels in hippocampi after blarcamesine administration (Welch’s t-test p = 0.0008). ANOVA analyses based on 6 animals per group confirmed these findings, by demonstrating that vehicle-treated KO2 mice had markedly lower levels of BDNF than vehicle-treated WT mice and they returned to WT levels after drug treatment (Fig. 2c).

### Blarcamesine yielded dose-dependent increases in S1R occupancy

Additional insight into the mechanisms by which blarcamesine improved key behavioral phenotypes and signaling pathway markers was obtained by analyzing the drug’s effects on S1R occupancy.

### PET/CT scanning

A two-tissue compartment model (2TCM) was successfully fitted to the 60-min dynamic mouse brain data in order to calculate the k3/k4 macro parameter. Data points were weighted by frame duration and blood volume was calculated for each animal. The whole blood image-derived arterial input function (IDIF) was described by a 3-exponential model and the chi value was used to determine best fit. The analyses of [^18^F]FTC-146 metabolism in 7-week-old WT mice showed that 16% of counts in plasma could be attributed to the parent radioligand 5 min post-dose (Fig. S1); the data also suggested an average fixed correction of 1:1.14 for whole blood counts to plasma. Model parameters and receptor occupancy calculations are summarized in Table 1.

**Table 1:** Calculated parameters from [^18^F]FTC-146 PET imaging including blarcamesine’s binding potential, total volume, volume of specific binding, S1R receptor occupancy (RO) and percent of injected dose per gram of tissue (%ID/g) in WT and *Fmr1* KO mice.

Dose group	Binding potential (k3/k4)	Total volume	Volume of specific binding	% RO	%ID/g
**WT**					
1 mg/kg PRE-084	0.78 ± 0.12	13.82 ± 1.19	6.01 ± 0.14	17.64 ± 6.57	7.64 ± 0.24
0 mg/kg blarcamesine (vehicle)	1.02 ± 0.17	15.27 ± 3.48	7.67 ± 1.81	N/A	7.75 ± 0.94
1 mg/kg blarcamesine	0.81 ± 0.11	13.64 ± 2.15	6.03 ± 0.68	14.30 ± 7.63	6.15 ± 0.94
10 mg/kg blarcamesine	0.34 ± 0.09	7.93 ± 1.82	2.02 ± 0.80	62.63 ± 10.23	3.39 ± 0.84
30 mg/kg blarcamesine	0.32 ± 0.11	8.16 ± 1.80	2.03 ± 0.92	64.44 ± 11.89	3.48 ± 0.65
***Fmr1* KO**					
0 mg/kg blarcamesine (vehicle)	0.88 ± 0.07	15.75 ± 2.24	7.33 ± 0.80	N/A	7.64 ± 1.38
1 mg/kg blarcamesine	0.85 ± 0.18	12.02 ± 0.21	5.23 ± 0.22	10.61 ± 5.25	5.27 ± 0.81
10 mg/kg blarcamesine	0.51 ± 0.17	9.34 ± 0.85	3.12 ± 0.97	41.15 ± 19.93	3.73 ± 0.83
30 mg/kg blarcamesine	0.31 ± 0.07	8.25 ± 0.66	1.93 ± 0.36	64.29 ± 8.56	3.21 ± 0.62

With exception of % RO, all values represent mean ± standard deviation.

Specifically, Table 1 depicts comparable 2-tissue model parameters, receptor occupancy (RO) and percent of injected dose per gram of tissue (%ID/g) calculations for WT and *Fmr1* KO mice. In these studies, blarcamesine oral (PO) dosing was 1 mg per kilogram of weight (mg/kg), 10 mg/kg, and 30 mg/kg, while S1R blocking with PRE-084 S1R was at a 1 mg/kg PO dose. No significant differences were found between blarcamesine’s RO in WT mice and KO mice. In Table 1, values are expressed as mean ± standard deviation, with the exception of % RO. Calculations of % RO are described in “Materials and methods” section.

**Figure 3:**
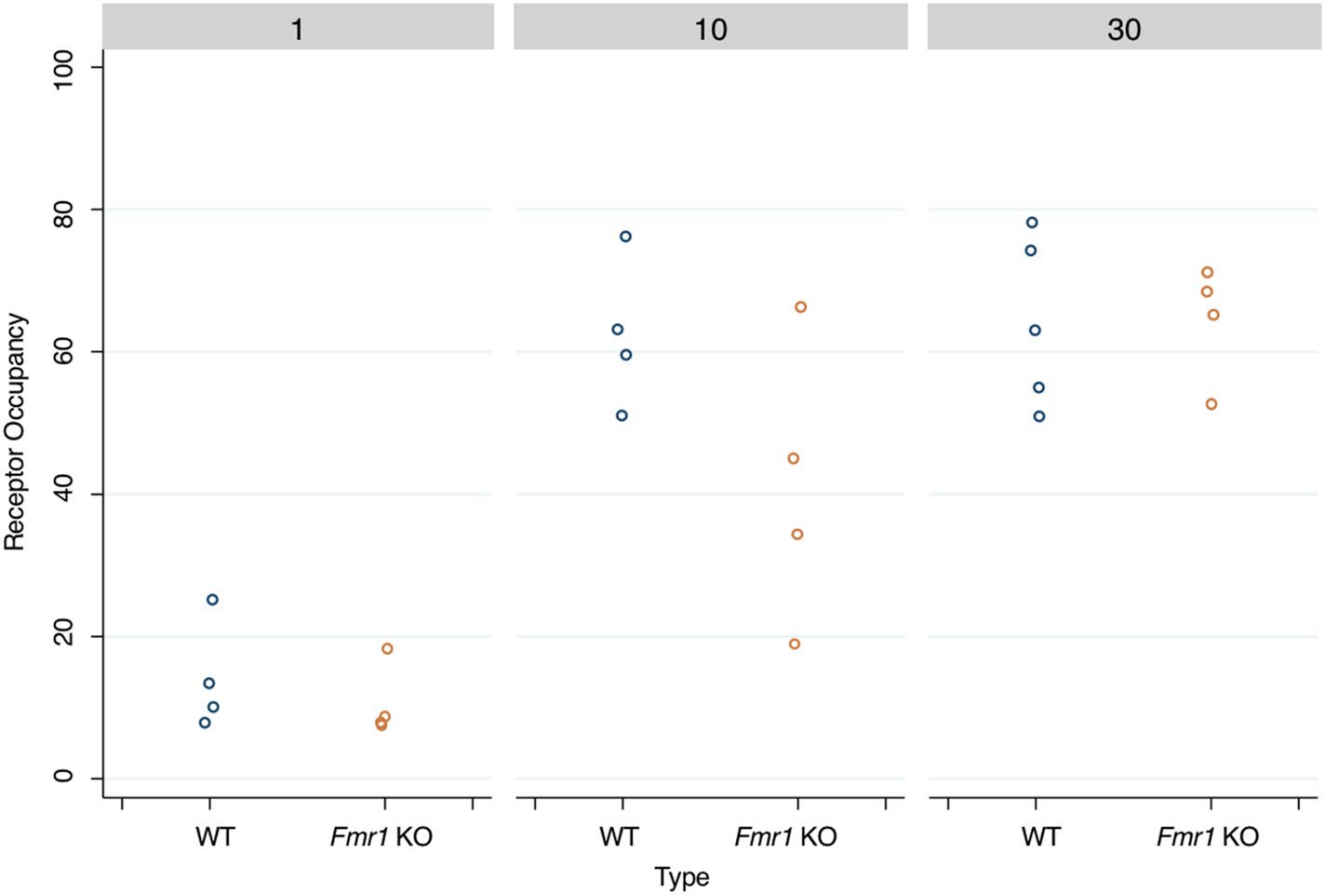
Effect of blarcamesine’s dose on S1R receptor occupancy (RO) in the whole brain using two-tissue compartmental modelling. Data points represent % RO of [^18^F]FTC-146 in each animal at each dose (1 mg/kg, 10 mg/kg and 30 mg/kg PO) in WT and *Fmr1* KO mice.

**Figure 4:**
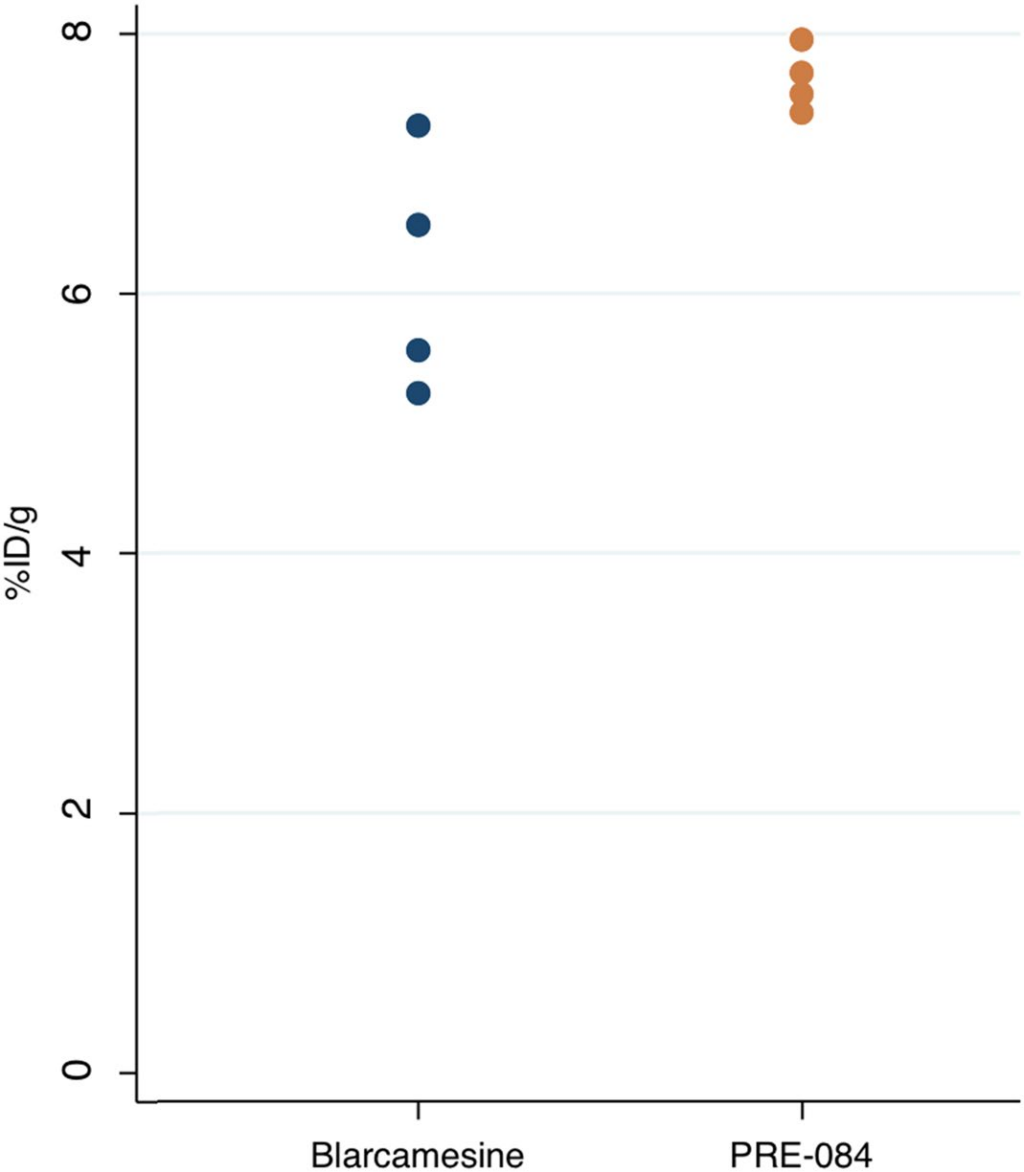
Comparing the blocking effectiveness of blarcamesine and PRE-084 at 1 mg/kg on [^18^F]FTC-146 binding to the S1R. Blarcamesine has a significantly higher blocking effectiveness demonstrated by lower %ID/g when compared to PRE-084 at the same dose (exact Wilcoxon test *p < 0.05).

S1R occupancy increased proportionally to the blarcamesine dose, with a plateau of 64% ± 9% at 30 mg/kg PO in WT mice and 64% ± 12% in *Fmr1* KO mice for the whole brain (Fig. 3, Table 1). When comparing k3/k4 parameter values in the absence of blarcamesine, no significant differences were observed between *Fmr1* KO and WT mice (0.88 ± 0.06 vs. 1.02 ± 0.17), suggesting that there were no discernable differences in the number of S1Rs present in the whole brain. For all outcome measures in the blarcamesine and control groups (k3/k4, total volume, volume of specific binding, % receptor occupancy and %ID/g), there was a significant decreasing trend across dose levels (all p < 0.001), but no effect of genotype (%ID/g p = 0.47, all other parameters p = 0.23). This data supports the notion that blarcamesine binds to the S1R receptor and that its occupancy can be imaged using the highly selecting S1R radiotracer, [^18^F]FTC-146. Moreover, at each dose, there was no effect of genotype on any calculated measure. This indicates that the amount of S1R present in *Fmr1* KO mice does not differ from WT when measured with [^18^F]FTC-146 PET imaging. PRE-084, an established reference S1R agonist^36^, was also administered PO at 1 mg/kg showing no significant difference to blarcamesine on any measured outcome (all p = 0.77) except with %ID/g, measured from 30 to 40 min, which showed significantly better blocking by blarcamesine (p = 0.029) (Table 1, Fig. 4).

### Ex vivo autoradiography

**Figure 5:**
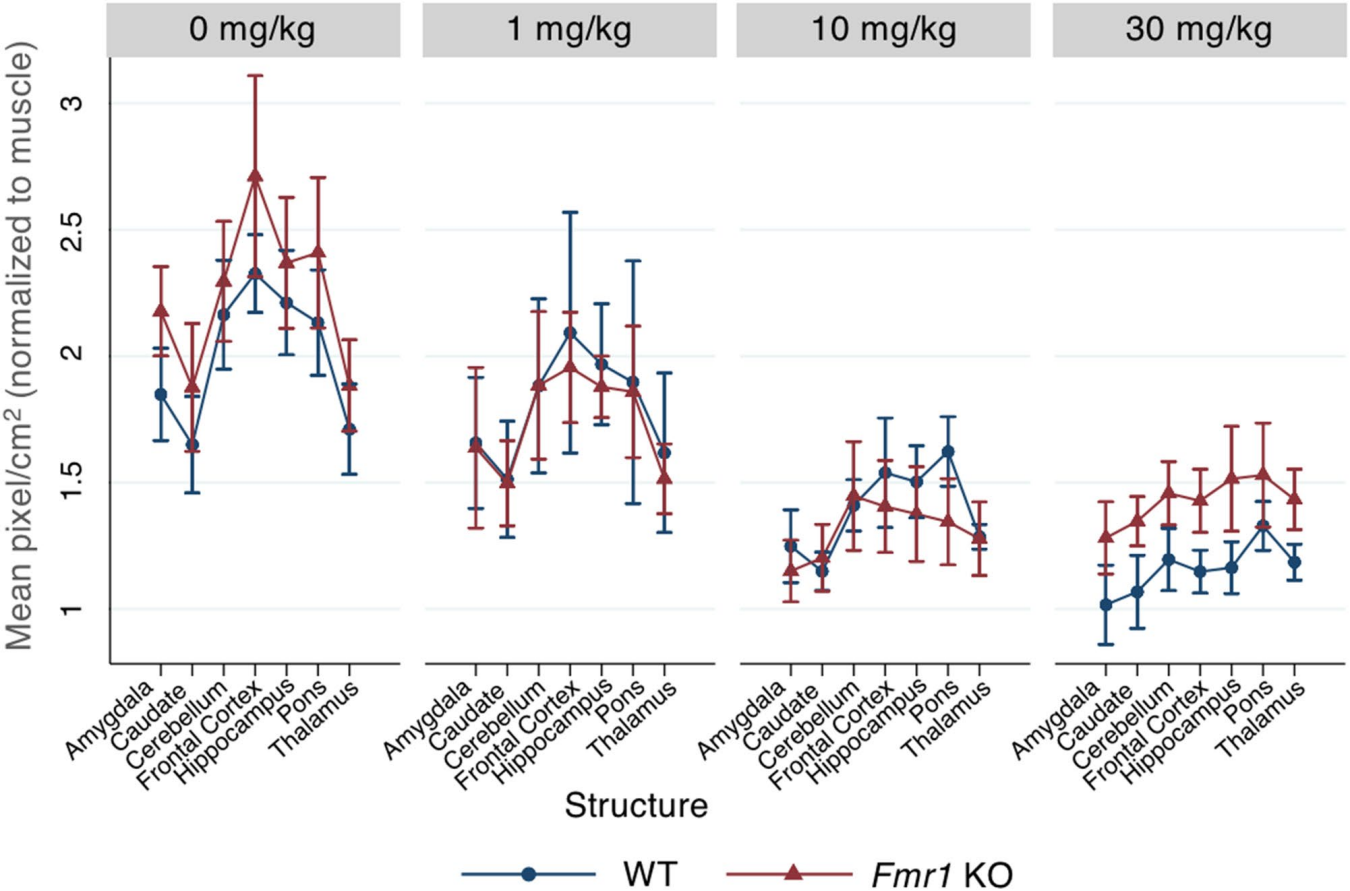
Effects of increasing blarcamesine dose on the binding of [^18^F]FTC-146 in different brain regions in WT and *Fmr1* KO mice via ex vivo autoradiography. Data points presented as mean pixel intensity normalized to the muscle (an internal control) within each animal and standard deviation. Ex vivo autoradiographic analyses of binding in frontal cortex, caudate, hippocampus, thalamus, amygdala, pons and cerebellum. Doses of blarcamesine were the same as used in the PET studies: 0, 1, 10 and 30 mg/kg PO.

**Figure 6:**
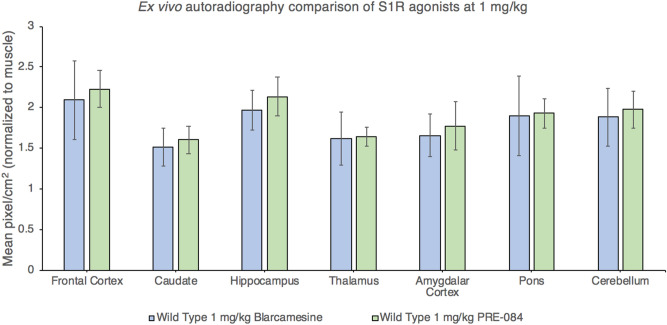
Ex vivo [^18^F]FTC-146 autoradiography comparison of blarcamesine and PRE-084 binding at 1 mg/kg PO in WT mice. Both S1R agonists showed similar S1R blocking in various brain regions in WT mice (N = 4–5 per group). Levels of binding were compared with those of control WT mouse. Error bars show standard deviation.

In order to further examine the uptake of [^18^F]FTC-146 in various brain regions at higher resolution, ex vivo autoradiography (ARG) was performed immediately after PET imaging in the frontal cortex, caudate, hippocampus, thalamus, amygdala, pons, and cerebellum. [^18^F]FTC-146 binding levels via ex vivo ARG varied significantly among brain structures (p < 0.001), particularly in the control and 1 mg/kg dose groups. There was a significant decreasing dose effect (p < 0.001), but no significant genotype effect (p = 0.15) (Fig. 5, Supplementary Fig. S2). Dosing at 1 mg/kg PO of either blarcamesine or PRE-084, a reference S1R agonist, exhibited no significant difference in S1R blocking in WT mice (p = 0.13) (Fig. 6).

## Discussion

Fragile X syndrome (FXS) is the most prevalent genetic form of intellectual disability and autism spectrum disorder^1,2^. As most neurodevelopmental disorders, FXS is considered a disorder of synaptic development and function^1,9^. Mouse models of FXS display a variety of cognitive and behavioral impairments of clinical relevance^9,40^. Despite advances in drug development using these experimental models, no pivotal trial in FXS has thus far been successful; therefore, this neurodevelopment disorder continues to have an unmet therapeutic need. Administration of blarcamesine to *Fmr1* KO2 mice for two weeks led to correction of two key neurobehavioral phenotypes and marked improvement of a third one. Moreover, a major neuronal signaling abnormality in mouse models of FXS, namely decreased BDNF levels, was restored to WT levels in the hippocampus of *Fmr1* KO2 mice. Since BDNF is a converging point of several synaptic regulators disrupted in FXS, these findings suggest that blarcamesine corrects *Fmr1* KO2 mouse behavioral phenotypes through multiple synaptic signaling mechanisms known to be affected by FMRP deficiency^9,31^. These dramatic effects of blarcamesine upon the *Fmr1* KO mouse phenotype can be explained by the drug’s dose-dependent level of S1R occupancy and the wide but variable brain regional distribution of these receptors^19^, which are not affected by *Fmr1* mutation, as demonstrated by the imaging component of the present study.

*Fmr1* mouse models of FXS are very informative since they replicate a wide range of molecular, anatomical, physiological, cognitive, and behavioral features of the disorder^9,40,52^. The tested behavioral paradigms covered three key aspects of FXS with implications for other neurodevelopmental disorders: cognitive impairment, ADHD features, and anxious and perseverative behaviors^1,2^. The fact that our investigation involved chronic administration, as opposed to acute dosing as in previous studies^53,54^, provides additional evidence in favor of the clinical use of blarcamesine. Since the behavioral paradigms reflect the involvement of multiple cortical and subcortical regions, their marked improvement by blarcamesine suggest widespread activation of S1Rs by the drug and modulation of multiple neural pathways. Indeed, the observation of normalization of BDNF levels after blarcamesine administration, in a brain region critical for cognition and behavior, is also a finding with important implications for FXS and other synaptic disorders^30^. BDNF is point of bidirectional convergence of several signaling pathways implicated in FXS pathogenesis, including PKA, PI3K/Akt/mTOR and MAPK/ERK, and plays key roles in neuronal and synaptic development as well as in the maintenance of circuitry^31,51^. As BDNF levels normalize without changes in pGSK-3β and Rac1 at the tested blarcamesine dose, these results suggest relative specificity in drug effects since the latter pathways are not directly linked to BDNF^31^. Experimental data suggests that FMRP and BDNF regulate each other and levels of BDNF modulate the FXS phenotype^31^. The recent demonstration of blarcamesine’s effect of enhancing autophagy in both animal and cellular models^16^ suggests that the drug may also play a role in correcting impaired autophagy and protein homeostasis in FXS^9^. *Fmr1* KO mice show multiple abnormalities in synaptic plasticity^9^, including compensatory homeostatic synaptic scaling^8^. The latter is a fundamental synaptic plasticity process that has been corrected, by administration of S1R agonists, in mouse models of other neurologic disorders^18^ and likely one of the mechanisms of blarcamesine’s action, in addition to stabilization of cellular bioenergetic and fostering neuronal homeostasis, all representing the wide range of functions of S1Rs^13–15^.

In the evaluation of novel compounds presumed to be centrally active, such as blarcamesine, it is necessary to confirm that the drug engages the targeted receptor within the central nervous system (CNS). PET imaging studies are useful tools in this effort because they provide a means to (1) demonstrate that the drug crosses the blood–brain barrier and (2) calculate the percentage of receptors that are occupied in vivo over time. Based on the results of the PET and ex vivo ARG analyses in the present study, S1R occupancy by blarcamesine was observed to be dose-dependent. It is shown that target engagement of S1Rs with blarcamesine is achieved already at relatively low doses with similar receptor occupancy profile to widely-used agonist PRE-084 at 1 mg/kg (Figs. 4, 6), thus providing a broader therapeutic window for S1R activation by this drug. Also, comparable S1R levels in *Fmr1* KO and WT mice support the notion of S1R preservation and the therapeutic potential of modulators of this receptor in FXS. The observed saturation of target occupancy before reaching 100% is likely due to the nature of the modulatory binding to the S1R by blarcamesine**.** A clinically-relevant component to this evaluation of receptor occupancy was evaluating blarcamesine’s oral administration used in the imaging studies, which demonstrated target engagement when administered via the route intended for human use. All PET scans were 60-min dynamic scans except for one scan which terminated at 55-min due to scanner error, but attenuation was not affected. In addition to demonstrating the adequacy of blarcamesine as a S1R agonist in the CNS through [^18^F]FTC-146 imaging, ex vivo ARG of multiple brain regions with the radioligand confirmed the wide distribution and region-specific binding of S1Rs. It also showed that there is no difference, at any blarcamesine dose, in regional S1R binding between WT and *Fmr1* KO mice. Since there is no reference region for the [^18^F]FTC-146, the overall mean of all structures was used as the reference region. In the post-scan ex vivo ARG, we observed that while no significant differences were observed between genotype groups from 0 to 10 mg doses, the WT group appears to begin to exhibit higher blocking than the *Fmr1* KO group at the highest blarcamesine dose. Perhaps due to limit of detection within our sample size, no significance was found; however, this possible genotype difference in the highest dose group is worth investigating in a larger sample size in the future. Another noticeable trend is that as the dose of blarcamesine increases, the differences between [^18^F]FTC-146 uptake in each structure decreases. This observation can be attributed to decreased S1R availability for radioligand binding due to an increase of receptor occupancy by blarcamesine in each structure. In Table 1, it is also notable that the 10 mg/kg *Fmr1* KO group has the highest standard deviation for %RO calculations. This may be due to the *Fmr1* KO mice requiring a higher dose to reach saturation compared to the WT mice, which are near saturation at 10 mg/kg. Receptor binding as a function of ligand concentration can be described by the Hill-Langmuir equation^55^, which reflects the degree of cooperativity between ligands. This equation describes the receptor occupancy range from 0% to saturation and includes an exponential phase within that range. The *Fmr1* KO mice may be in the exponential phase at 10 mg/kg, therefore, small variations in effective ligand concentration will thus lead to large variations in occupancy, as reflected in the data. At 30 mg/kg, once saturation is reached, the datapoints for both groups of mice are very similar. As mentioned in our methods section, for the blarcamesine-dosed groups, one animal from each dose-group were scanned at a time, for a total of 4 animals per scan, to uphold the scientific rigor of our scanning. This design allows the measurement of animals from each dosing group to be taken on multiple scan days over the course of several weeks accounting for variations to be included between scans, radiotracer productions and different animal cages. Thus, the implemented design resulted in higher standard deviations due to accumulated data for each cohort being collected over multiple scans, rather than a single scan. Similarly, in Table 1 and Fig. 4, a larger standard deviation for the %ID/g values was observed in the 1 mg/kg blarcamesine WT dose group (animals were separately imaged over several weeks) when compared to the 1 mg/kg PRE-084 WT dosed group (animals were imaged in the same scan). Overall, through all parameters examined, our findings further support the notion that, while the measured amount of [^18^F]FTC-146 bound to S1Rs changes throughout the WT brain, there were no observed changes between WT and *Fmr1* KO mice.

Altogether, these neurobehavioral, biochemical, and imaging data demonstrate that corresponding doses of blarcamesine that yield measurable receptor occupancy are effective for substantially correcting key synaptic and behavioral phenotypes in *Fmr1* KO mice. Our data also suggest that these positive effects are mediated by S1R activation in multiple brain regions, where blarcamesine binds to the receptor in a dose-dependent and genotype-independent manner. Limitations of the present study include the relatively small number of behavioral paradigms examined, some with limited range of responses; the range and duration of drug dosing; evaluation of BDNF and other signaling molecules in a single brain region; and the use of two different *Fmr1* KO models in the neurobehavioral and imaging studies. Follow up dose–response studies can be performed to determine the point of saturation in the *Fmr1* KO mice and examine the effects of different doses on positive responses and their variability in multiple brain regions relevant to the FXS phenotype (e.g., neocortex). Endpoints in these investigations ought to include indices of activation of BDNF-related signaling pathways known to be affected in FXS and *Fmr1* mouse models. A shortcoming of using mice in the imaging studies was the need of a separate cohort of mice to the one subjected to PET imaging to determine both plasma:whole blood ratio and metabolism of [^18^F]FTC-146 over time. This was done because, in order to obtain metabolic profiles from the same mice being scanned, a large amount of blood (~ 25–35% of total blood volume) would have been needed with the resulting perturbation in animal physiology. For these studies, WT mice were used because there was no expected change in metabolism between WT and *Fmr1* KO mice. Furthermore, a separate group of WT and *Fmr1* KO mice was used as the control for both the PET and autoradiography studies, rather than imaging a baseline in each mouse.

In conclusion, the present findings confirm the dose-dependent receptor occupancy of the S1R with blarcamesine and, combined with the therapeutic response observed at low doses in the tested preclinical model, emphasize the viability of S1R as a therapeutic target in FXS and the clinical potential of blarcamesine in FXS and other neurological disorders. Indeed, pre-clinical studies in a mouse model of Rett syndrome showed similar positive effects on multiple clinically relevant neurobehavioral phenotypes^21^. Furthermore, clinical efficacy was demonstrated in a placebo-controlled Phase 2 study in Rett syndrome (NCT03758924) and previously in a smaller PK cohort of patients with this neurodevelopmental disorder^39^, as well as significant cognitive improvements in a Phase 2 trial in Parkinson’s disease dementia (NCT03774459). Late-stage clinical studies of blarcamesine in adult and pediatric patients with Rett syndrome (NCT03941444, NCT04304482) and Alzheimer’s disease (NCT02756858, NCT03790709) are currently ongoing. Continued findings from these clinical studies with blarcamesine, combined with the presented data strengthens the rationale for potentially a dependable and effective treatment strategy for FXS and other neurological disorders targeting the S1R with blarcamesine.

## Materials and methods

The study was divided into two parts, the first of which involved behavioral and biochemical assays to characterize the effect of blarcamesine in reversing the murine FXS phenotype. The second component, also carried out in mice, focused on determining the drug’s S1R receptor occupancy by PET and S1R distribution by ex vivo autoradiography. All animal studies were performed in accordance to Animal Research: Reporting of In Vivo Experiments (ARRIVE) guidelines. The behavioral and biochemical assay studies were carried out in accordance with the guidelines and regulations of the United Kingdom Animals (Scientific Procedures) Act of 1986. The PET imaging and receptor occupancy studies were carried out in accordance with the guidelines and regulations of Stanford University’s Institutional Animal Care and Use Committee (IACUC).

### Behavioral and cell signaling analyses

#### Animals

For both the behavioral and biochemical assessments, experiments were conducted in accordance with the United Kingdom Animals (Scientific Procedures) Act of 1986. *Fmr1* KO2 mice^40^ and wild type (WT) littermates, which were generated on a C57BL/6J background and repeatedly backcrossed onto a C57BL/6J background for more than eight generations, were provided by Professor David Nelson (Baylor College of Medicine, Houston, TX, USA) and the FRAXA Research Foundation. Mice were housed in commercial plastic cages on a ventilated rack system without enrichment, in groups (4–6 per cage). All animals were provided with ad libitum food and water and maintained on a 12 h light/dark cycle in a temperature-controlled environment (21 ± 1 °C). All studies were conducted on male mice. In contrast with the *Fmr1* KO mouse^41^, the first murine model of FXS, the more recently developed *Fmr1* KO2 mouse^40^, is characterized by no expression of *Fmr1* mRNA (the *Fmr1* KO mouse expresses up to 27% of WT brain *Fmr1* mRNA levels^40^). Both mouse models do not express FMRP and show no substantial phenotypical differences^56^.

#### Drug treatment

Blarcamesine was administered to 2-month-old animals twice daily at a dose of 1 mg/kg IP for a total of 14 days. Saline served as vehicle and control. For behavioral studies, four dose groups (N = 10 mice per group, 40 mice total) were included: 2 groups of WT mice given either blarcamesine or saline and 2 groups of *Fmr1* KO2 mice given either blarcamesine or saline. For the cellular assays assay, four dose groups (pGSK-3β: N=10 mice per group, Rac1: N=10 mice per group, BDNF: N=6 mice per group, BDNF: N=6 mice per group pERK: N = 7, 28 mice total, BDNF: N = 6 mice per group, 24 total) were included: 2 groups of WT mice given either blarcamesine or saline and 2 groups of *Fmr1* KO2 mice given either blarcamesine or saline. Animals were inspected for changes in general appearance that might occur following a single dose, prior to the onset of chronic dosing. Items monitored in these tolerability assessments included coat appearance, piloerection, eye conditions (runny eyes or porphyria, ptosis), gait, tremor, tail tone, and reactivity to handling.

#### Behavior

Behavioral testing was conducted during the light phase at 2 months of age, with experimenter’s blind to genotype and drug treatment. Mice were tested with one of three behavioral tasks (open field, contextual fear conditioning, or marble burying) on each experimental day; each behavioral test was separated by 3 days. Prior to behavioral testing, mice were randomly assigned to treatment groups. Apparatuses were cleaned with moist and dry tissues before testing each mouse, in order to create a low but constant background mouse odor for all experimental subjects. Behavioral tests served to characterize efficacy-related key endpoints of relevance to the FXS phenotype and performed as previously published^1,2,57,58^ (see Supplementary Materials for further details): Open field test^58^ (anxiety, hyperactivity, habituation to a novel environment), contextual fear conditioning^58^ (associative learning), and marble-burying^48^ (anxiety, perseverative behavior).

#### Cell signaling analyses

Western blot and ELISA assays for measuring (1) activated glycogen synthase kinase 3 beta (pGSK-3β), (2) Ras-related C3 botulinum toxin substrate 1 (Rac1) expression, and (3) brain-derived neurotrophic factor (BDNF) expression was conducted in hippocampal homogenates. This brain region was selected because of its role in the abovementioned behavioral paradigms and other key phenotypes in FXS mouse models^59^. Hippocampi were collected from mice sacrificed by CO_2_ followed by cervical dislocation. Samples were frozen on dry ice and stored at − 70 °C until use. The aforementioned assays were performed as previously published^50,57,60^ with more details in the Supplementary Materials.

#### Statistical analysis

Data obtained from behavioral tests and molecular assays were first characterized in terms of descriptive features, with a focus on distribution. The Shapiro–Wilk test of normality was applied to each dataset, complemented by the Kolmogorov–Smirnov test for those datasets with many identical values. Equality of variances was assessed by the Levene’s, Brown-Forsythe’s, and Bartlett’s tests. For all analyses involving groups without normal distribution or equal variances, additional nonparametric tests were performed. For all these tests, p-value less than 0.05 was considered statistically significant. Analyses were conducted using SPSS version 25 (IBM, Armonk, NY, USA), as well as several online calculators including Statistics Kingdom, Social Science Statistics, Statology, and iCalcu.com. Further details on this analysis can be found in the Supplementary Materials.

### Imaging studies

#### Animals

Animal experiments were approved by Stanford’s IACUC. Experiments were carried out using adult (7-week-old) male mice weighing 23–30 g (WT: FVB.129P2-Pde6b + Tyr^c-ch^/AntJ; *Fmr1* KO: FVB.129P2-Pde6b + Tyr^c-ch^ *Fmr1*^tm1Cgr^/J, both from the Jackson Laboratory (Bar Harbor, ME, USA). These *Fmr1* KO mice corresponded to the first murine model of FXS^41^, described in preceding sections. Animals had access to food and water ad libitum and were kept under a 12 h light/dark cycle in cages of 3–5 mice. The animals were included in the study if they received successful administration of blarcamesine and radiotracer [^18^F]FTC-146, and completed PET scan without motion. Animals with failed radiotracer injections or who had motion during the scan due to scanning beds shifting mid-scan were excluded from this dataset.

#### General

Unless stated otherwise, all compounds and chemicals were purchased from commercial sources and used without modification. PET imaging was performed using a micro-PET/computed tomography (CT) or D-PET equipped with cobalt point source (Inveon; Siemens Medical Solutions Inc, Tarrytown, NY, USA). Attenuation correction was applied to each dataset from the CT or cobalt transmission images. Frames were reconstructed using three-dimension ordered-subset expectation maximization (3DOSEM). FTC-146 tosylate precursor and reference standard were both synthesized under contract from Albany Molecular Research, Inc (Albany, NY, USA). Blarcamesine was manufactured and provided by Anavex Life Sciences Corp. (New York, NY, USA).

#### Radiochemistry

[^18^F]FTC-146 was synthesized as previously reported^44^. At the end of [^18^F]FTC-146 production, molar radioactivity was 12.8 ± 5.7 Curie/micromole (Ci/µmol) (474 ± 211 gigabecquerel (GBq/µmol) and radiochemical purity was 91–94%.

#### Drug treatment

Blarcamesine was administered orally to 7-week-old animals in 4 dose groups (0, 1, 10, 30 mg/kg PO; N = 4–5 mice per treatment group); PRE-084 (Cayman Chemicals, Ann Arbor, MI, USA), another S1R agonist^36^, was administered orally in a single dose group (1 mg/kg; N = 4 mice per treatment group).

#### PET/CT scanning

To uphold scientific rigor for each PET scan, 4 mice were scanned at a time in a custom “hotel” PET bed with one mouse from each blarcamesine dosing group or solely PRE-084 mice. In some cases, multiple mice from a single blarcamesine dosing group were scanned together if a dosing group needed to be repeated due to failed injection or motion in the scanning bed. Mice were anesthetized using humidified, oxygen-enriched isoflurane gas (5.0% for induction and 1.5–2.5% for maintenance) 20 min after administration of blarcamesine, then tail vein catheters were inserted. After 60 min post-drug delivery, a dynamic PET scan of 60 min (frames: 60 × 3 s, 12 × 1 min, 3 × 5 min, 3 × 10 min) was commenced just before a bolus of [^18^F]FTC-146 (210 ± 22 µCi, 7.77 ± 0.81 MBq) was injected intravenously.

#### Ex vivo autoradiography

Following the PET scan, the mice were perfused with 30 mL PBS and brains and leg muscle were collected, frozen on dry ice in Optimal Cutting Temperature compound (Tissue-Tek, Sakura Finetek USA Inc., Torrance, CA, USA) and sectioned in the coronal plane on a cryotome (Leica 3050S, Leica Biosystems, Wetzlar, Germany) at 20 µm for ex vivo autoradiography. Collected brain regions included: frontal cortex (between bregma + 3.33 and + 2.43), caudate (between bregma + 1.23 and + 0.23), hippocampus, thalamus and amygdala (between bregma − 1.47 and − 2.07), pons (between bregma − 3.97 and − 4.57), cerebellum (between bregma − 5.67 and − 6.97), and thigh muscle for normalization. Sections were incubated on a phosphor-storage screen (GE Healthcare, Chicago, IL, USA) for 20–24 h and imaged using a GE Healthcare Typhoon Trio (GE Healthcare, Chicago, IL, USA).

#### Radiometabolite analysis

For our protocol for radiometabolite analysis, please see Supplementary Materials.

#### Data analysis

PET images were analyzed by drawing 3-dimensional regions around the whole brain. A two-tissue compartment (2TCM) model was used to fit the measured time activity curve (TAC) for brain using PMOD software version 3.7 (PMOD Technologies LLC, Zurich, Switzerland) and to calculate k3/k4 also known as the binding potential for the specific binding at equilibrium in relation to non-displaceable binding (BP_ND_)^61,62^, which was used as binding potential (BP) for the subsequent receptor occupancy calculations, as previously described^62^. To obtain the arterial whole blood input function, an imaged-derived input function (IDIF) was determined by drawing the volume of interest over the left heart ventricle representing the highest pool of radiotracer in the blood. Both the plasma:whole blood ratio and the % intact parent [^18^F]FTC-146 over time were incorporated into the PMOD software to generate the 2TCM model to estimate BP, represented as k3/k4, and was used for receptor occupancy calculations. Calculation of the percent of injected dose per gram (%ID/g) was also used to assess target engagement of blarcamesine. For %ID/g calculations, a time period of 30–40 min was examined. To analyze ex vivo autoradiography images, ImageJ 1.48v^63^ was used to define regions of interest and all structures were normalized to muscle. Three samples for each region of interest was collected from each mouse and averaged. These studies were not blinded as one person performed all dosing, scanning, PET and ex vivo ARG image analysis.

#### Statistical analysis

WT (N = 18) and *Fmr1* KO (N = 17) mice in groups at each of four blarcamesine dosage levels (0, 1, 10, 30 mg/kg) were scanned via PET/CT or D-PET. Using 2TCM as described above, the following parameters were calculated: binding potential (k3/k4), specific volume (V_s_) bound, total volume bound (V_t_). An additional four WT mice were given 1 mg/kg PRE-084 were analyzed separately. Due to the between-subjects design, receptor occupancy at dose d, defined as (BP(0) − BP(d))/BP(0) × 100 could not be calculated per-individual. As an approximation, the median value of BP(0) for type of animal was used and any occupancy value ≤ 0 was replaced with the lowest observed positive value.

The effect of dose was tested with a nonparametric linear-by-linear association test of trend stratified by genotype; the effect of genotype was tested by a Wilcoxon rank sum (Mann–Whitney) test stratified by dose. Comparison of drugs was also done by the Wilcoxon rank sum test.

Binding of [^18^F]FTC-146 was evaluated via %ID/g from 30 to 40 min post-injection for each dose of blarcamesine or PRE-084. The effects of genotype and blarcamesine dose on %ID/g were tested with a regression of %ID/g on genotype and dose. The effect of drug type when comparing blarcamesine and PRE-084 on %ID/g (in 4 WT animals per each drug at dose 1 mg/kg) was tested with an exact Wilcoxon test.

To assess binding of [^18^F]FTC-146 varying concentrations of blarcamesine or PRE-084 in post-scan ex vivo autoradiography (ARG), the regions of interest were hand-drawn in Image J, in triplicate for each region (3 slices per region). The mean pixel intensity values for each brain region (frontal cortex, caudate, hippocampus, thalamus, amygdala cortex, pons and cerebellum) were averaged. The effects of genotype, blarcamesine dose and brain structure on ARG were tested with a generalized linear regression with log link of ARG on genotype, dose and location, adjusted for clustering within animal. Since there is no established reference brain region for [^18^F]FTC-146, the overall mean across the structures of interest (adjusted for dose and type) was used as the reference value to compare tracer uptake among these structures.

The effect of drug type when comparing blarcamesine and PRE-084 via ARG (in 4 WT animals per each drug at dose 1 mg/kg) was tested with van Elteren’s test (stratified Wilcoxon rank sum test) using neural structures as strata.

Statistical analyses were done using R version 3.6.3 and package “coin” version 1.3-1. For all tests, a p-value less than 0.05 was considered statistically significant. Given the small sample and exploratory nature of this study, no correction for multiple testing was done.

## Supplementary Information

Supplementary Information.
