# Supplementary material for: Effects of the sigma-1 receptor agonist blarcamesine in a murine model of fragile X syndrome: neurobehavioral phenotypes and receptor occupancy
Source: Sci Rep. 2021 Aug 25;11:17150. doi: 10.1038/s41598-021-94079-7 (PMC8387417; doi:10.1038/s41598-021-94079-7)
Supplement: Supplementary file 1 — Supplementary Information. [file 41598_2021_94079_MOESM1_ESM.pdf]

Electronic Supplementary Material

Effects of the Sigma-1 Receptor Agonist Blarcamesine in a Murine Model of Fragile X Syndrome: Neurobehavioral Phenotypes and Receptor Occupancy

Journal: Scientific Reports

Samantha T. Reyes[1], Robert M.J. Deacon[2,3,4], Scarlett G. Guo[1], Francisco J. Altimiras[2,5], Jessa B. Castillo[1], Berend van der Wildt[1], Aimara P. Morales[1], Jun Hyung Park[1], Daniel Klamer[6], Jarrett Rosenberg[1], Lindsay M. Oberman[7], Nell Rebowe[6], Jeffrey Sprouse[6], Christopher U. Missling[6], Christopher R. McCurdy[8], Patricia Cogram[2,3,4], Walter E. Kaufmann[6,9*] and Frederick T. Chin[1*]

[1]Stanford University, Department of Radiology, Stanford, CA 94305, USA
[2]FRAXA-DVI, FRAXA, Santiago Chile
[3]IEB, Faculty of Science, University of Chile, Santiago, Chile
[4]Fraunhofer Chile Research, Center for Systems Biotechnology, Santiago, Chile
[5]Faculty of Engineering and Business, Universidad de las Américas, Santiago, Chile.
[6]Anavex Life Sciences Corp., New York, NY 10019, USA
[7]Center for Neuroscience & Regenerative Medicine, Uniformed Services University of the Health Sciences, Bethesda, MD
[8]Department of Medicinal Chemistry, College of Pharmacy, University of Florida, Gainesville, FL, 32610, USA
[9]Department of Human Genetics, Emory University School of Medicine, Atlanta, GA 30322, USA

***Correspondence to:**

Frederick T. Chin, Ph.D.
Email:chinf@stanford.edu

and

Walter E. Kaufmann, M.D.
E-mail: walter.e.kaufmann@emory.edu

Key Words: fragile X syndrome, Fmr1 KO, Fmr1 KO2, Sigma-1, blarcamesine, ANAVEX2-73, PRE-084, [18F]FTC-146, Positron Emission Tomography, cell signaling, mouse

Supplementary Materials and Methods:

Behavioral and Cell Signaling

Behavioral Tests

1. Open field test. The open-field assay was performed using an automated system including a VersaMax activity monitor chamber with the associated VersaDat software (Accuscan Instruments, Columbus, OH, USA). Mice were brought to the experimental room 20 min before testing. A mouse was placed into a corner square facing the corner and observed for 3 min. The number of squares crossed in that period of time was considered a measure of total distance traveled [45].

2. Contextual fear conditioning test. Standard procedures were used throughout. Training began by placing an animal in a novel environment (dark chamber), providing an aversive stimulus (a 1-sec electric shock, 0.2 mA, to the paws), and then removing it (conditioning chamber from Kinder Scientific, Poway, CA, USA). Freezing behavior upon return to the same environment, defined as the complete lack of motion for a minimum of 0.75 sec, was measured as percentage of total period of observation. The test is considered a species-specific response to fear and an associative learning task [45].

3. Marble burying test. Transparent plastic cages were filled with a 10 cm deep layer of sawdust on top of which 10 glass marbles were placed in two rows. Each animal was left undisturbed in such a cage for 30 min, after which the number of marbles buried to at least 2/3 of their depth was recorded [46].

Cell Signaling analyses

1. Activated glycogen synthase kinase 3 beta (pGSK-3β) and Ras-related C3 botulinum toxin substrate 1 (Rac1) expression. These signaling molecules were measured by Western blotting as reported [44]. For each sample, 30 µg of lysed protein samples was run on 12% polyacrylamide gels, blotted onto nitrocellulose membranes, and probed with anti-phosphospecific antibodies against GSK-3β (tyr216/tyr279; Sigma-Aldrich, St. Louis, MO, USA) or an anti-Rac1 antibody (Abcam, Cambridge, MA, USA). pGSK-3β and Rac1 levels were normalized to protein content and to levels of reference cytoskeletal proteins (β–actin for pGSK-3β, α–tubulin for Rac1), in the same sample. Levels were expressed as % of change with respect to basal conditions, considering basal levels (WT on vehicle) as 100%.

2. Brain-derived neurotrophic factor (BDNF) expression. BDNF levels were determined using a BDNF enzyme-linked immunosorbent assay (ELISA Quantikine human BDNF kit; R&D Systems, Abingdon, UK) as described previously [49].

Statistical analysis. Data obtained from behavioral tests and molecular assays were first characterized in terms of descriptive features, with a focus on distribution. The Shapiro-Wilk test of normality was applied to each dataset, complemented by the Kolmogorov-Smirnov test for those datasets with many identical values. Equality of variances was assessed by the Levene's, Brown-Forsythe's, and Bartlett's tests. These tests determined that, with exception of values for the WT groups on the marble burying test, all data were distributed normally although there were some trend-level significant differences in variances. The first group of analyses aimed at

identifying blarcamesine's efficacy by comparing drug-treated with vehicle-treated *Fmr1* KO2 groups on each behavioral or molecular assays. For this purpose, we used concurrently two types of independent two-sample t-tests; the Welch's t-test for mouse groups with trend level (p=0.05-0.10) F-test for equal variance and the Student's t-test for all the others. Once a significant difference was found, a two-way univariate ANOVA with genotype and treatment as between-subject factors, followed by a Tukey's post-hoc test, evaluated the differences between drug-treated and vehicle-treated WT and *Fmr1* KO2 mice. These analyses intended also to confirm that the phenotype of vehicle-treated *Fmr1* KO2 was abnormal. For all analyses involving groups without normal distribution or equal variances, additional nonparametric tests were performed. For all these tests, p-value less than 0.05 was considered statistically significant. Analyses were conducted using SPSS version 25 (IBM, Armonk, NY, USA), as well as several online calculators including Statistics Kingdom, Social Science Statistics, Statology, and iCalcu.com.

Imaging Studies

Radiometabolite analysis. An additional group of WT mice (age 57 days; N=6; no blarcamesine treatment) were used to derive the whole blood to plasma ratio and parent fraction for metabolite correction of the input function. Tail vein catheters were similarly inserted and the mice were injected intravenously with a bolus of [^{18}F]FTC-146 (780±214 µCi, 28.86±7.92 MBq). Arterial blood was collected from the left ventricle heart pool at 1, 5, 15, 30 and 60 min (N=1-2 per time point) and placed into heparinized tubes. Immediately after collection of arterial blood (0.3-0.5 mL), 20 µL of blood was pipetted into a gamma counting tube and the remaining arterial blood sample was spun down in a centrifuge (5 min, 1100 g). After centrifugation, the plasma supernatant (20 µL) was then pipetted into a separate gamma counting tube, and both whole blood and plasma aliquots were placed in a gamma counter (Hidex AMG, Turku, Finland)) to measure whole blood:plasma ratios for subsequent kinetic modeling correction. Plasma and ice-cold acetonitrile (1:1) were thoroughly mixed and spun down (5 min, 9500 g), with the resulting pellet retained for calculating extraction efficiency. Lastly, equal parts of plasma:acetonitrile mixture and ice-cold water were thoroughly mixed and stored on ice prior to HPLC analysis. Acetonitrile samples were injected to separate [^{18}F]FTC-146 parent and metabolite compounds using a radio-HPLC (Agilent 1200, Agilent Technologies, Santa Clara, CA, USA). The respective radioactive fractions were collected based on the radiochromatogram and measured in a gamma counter.

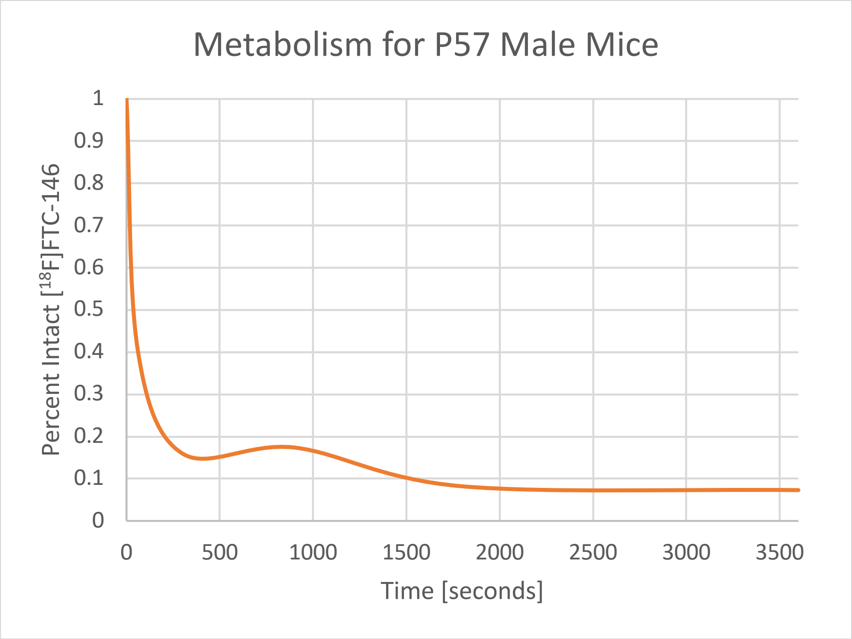

Figure S1. Metabolism of [^{18}F]FTC-146 in the blood over one hour. Parent-tracer intact in plasma calculated by gamma counting of plasma versus whole blood in an equal volume aliquot.

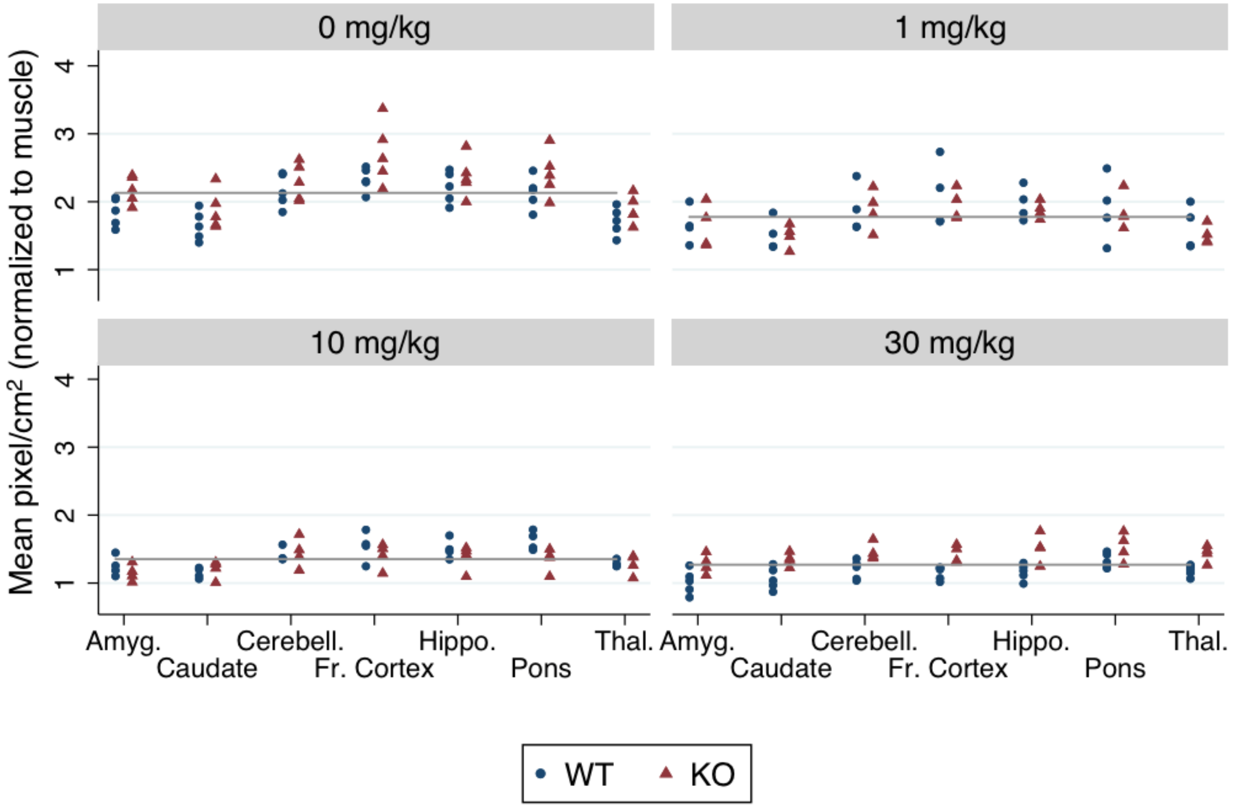

Figure S2. The effects of increasing blarcamesine dose on [^{18}F]FTC-146 in different brain regions in WT and *Fmr1* KO, comparing to grand mean uptake value of all structures. Data points presented as mean pixel intensity after normalization to internal control (muscle) and standard deviation. Since there is no established reference brain region for [^{18}F]FTC-146, the overall mean across the structures of interest (adjusted for dose and type) was used as the reference value, shown as a horizontal line on the figure) to compare tracer uptake among these structures. *Ex vivo* autoradiographic analyses of binding in frontal cortex, caudate, hippocampus, thalamus, amygdala, pons and cerebellum. Doses of blarcamesine were the same as used in the PET studies: 0, 1, 10 and 30 mg/kg PO.
